# Crystal structure of ethyl 2′′,3-dioxo-7′,7a’-di­hydro-1′*H*,3*H*,3′*H*-di­spiro[benzo[*b*]thio­phene-2,6′-pyrrolo­[1,2-*c*]thia­zole-5′,3′′-indoline]-7′-carboxyl­ate

**DOI:** 10.1107/S2056989015002030

**Published:** 2015-02-07

**Authors:** M. P. Savithri, M. Suresh, R. Raghunathan, R. Raja, A. SubbiahPandi

**Affiliations:** a Department of Physics, Queen Mary’s College (Autonomous), Chennai 600 004, India; b Department of Organic Chemistry, University of Madras, Guindy Campus, Chennai 600 025, India; c Department of Physics, Presidency College (Autonomous), Chennai 600 005, India

**Keywords:** crystal structure, di­spiro, benzo­thio­phene, thia­zole, pyrrolidine, indoline, hydrogen bonds, inversion dimers

## Abstract

In the title compound, C_23_H_20_N_2_O_4_S_2_, the central pyrrolidine ring adopts an envelope conformation with the spiro C atom, shared with the benzo­thio­phene ring system, as the flap. The thia­zole ring has a twisted conformation on the S—C bond, where the C atom is that closest to methine C atom. The mean planes of the benzo­thio­phene and indoline ring systems are inclined to the mean plane of the central pyrrolidine ring by 82.75 (8) and 80.03 (8)°, respectively, and to each other by 61.49 (6)°. In the crystal, mol­ecules are linked *via* pairs of N—H⋯O hydrogen bonds, forming inversion dimers with an *R*_2_^2^(8) ring motif. The dimers are linked *via* C—H⋯O and C—H⋯N hydrogen bonds, forming a three-dimensional structure. The eth­oxy­carbonyl group is disordered over two orientations, with an occupancy ratio of 0.717 (12):0.283 (12).

## Related literature   

For the biological activity of spiro-pyrrolidine derivatives, see: Obniska *et al.* (2003 ▸); Peddi *et al.* (2004 ▸); Zapf *et al.* (2011 ▸); Stylianakis *et al.* (2003 ▸); Waldmann (1995 ▸); Suzuki *et al.* (1994 ▸); Huryn *et al.* (1991 ▸). For a related structure, see: Narayanan *et al.* (2013 ▸).
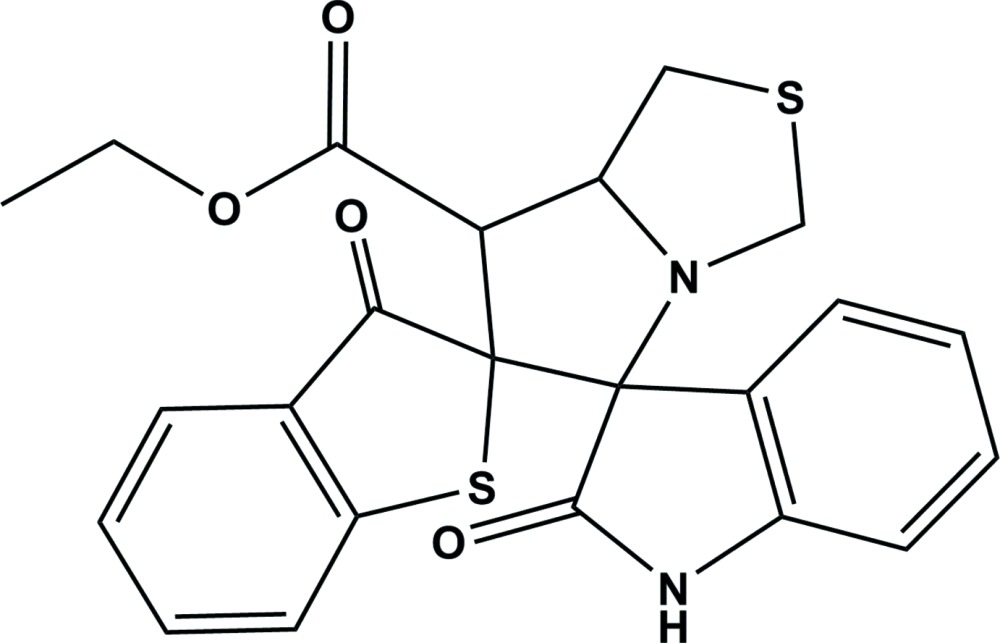


## Experimental   

### Crystal data  

C_23_H_20_N_2_O_4_S_2_
*M* *_r_* = 452.53
Monoclinic, 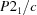

*a* = 11.8894 (5) Å
*b* = 10.2181 (4) Å
*c* = 17.5044 (8) Å
β = 97.991 (2)°
*V* = 2105.91 (15) Å^3^
*Z* = 4
Mo *K*α radiation
μ = 0.29 mm^−1^
*T* = 293 K
0.35 × 0.30 × 0.30 mm

### Data collection  

Bruker Kappa APEXII CCD diffractometer
Absorption correction: multi-scan (*SADABS*; Bruker, 2004 ▸) *T*_min_ = 0.906, *T*_max_ = 0.919
19635 measured reflections
3706 independent reflections
3169 reflections with *I* > 2σ(*I*)
*R*_int_ = 0.027

### Refinement  

*R*[*F* ^2^ > 2σ(*F* ^2^)] = 0.032
*wR*(*F* ^2^) = 0.088
*S* = 0.98
3705 reflections
312 parameters
65 restraints
H atoms treated by a mixture of independent and constrained refinement
Δρ_max_ = 0.26 e Å^−3^
Δρ_min_ = −0.27 e Å^−3^

### 

Data collection: *APEX2* (Bruker, 2004 ▸); cell refinement: *APEX2* and *SAINT* (Bruker, 2004 ▸); data reduction: *SAINT* and *XPREP* (Bruker, 2004 ▸); program(s) used to solve structure: *SHELXS97* (Sheldrick, 2008 ▸); program(s) used to refine structure: *SHELXL97* (Sheldrick, 2008 ▸, 2015 ▸); molecular graphics: *ORTEP-3 for Windows* (Farrugia, 2012 ▸); software used to prepare material for publication: *SHELXL97* and *PLATON* (Spek, 2009 ▸).

## Supplementary Material

Crystal structure: contains datablock(s) global, I. DOI: 10.1107/S2056989015002030/su5067sup1.cif

Structure factors: contains datablock(s) I. DOI: 10.1107/S2056989015002030/su5067Isup2.hkl

**Fig. 1.**
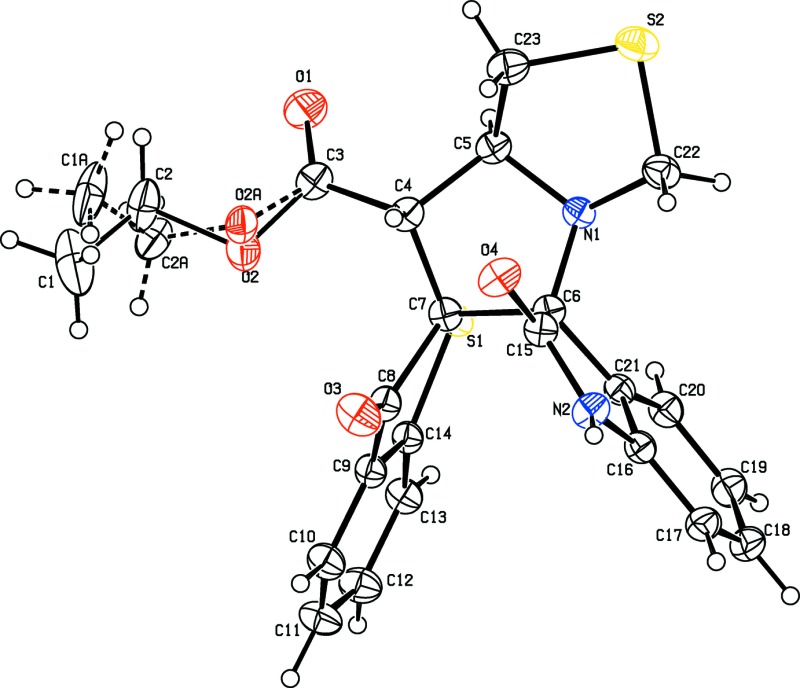
The molecular structure of the title compound with the atom numbering scheme. Displacement ellipsoids are drawn at the 30% probability level. The dashed lines represent the bonds involving the minor component of the disordered ethyl carboxylate unit.

**Fig. 2.**
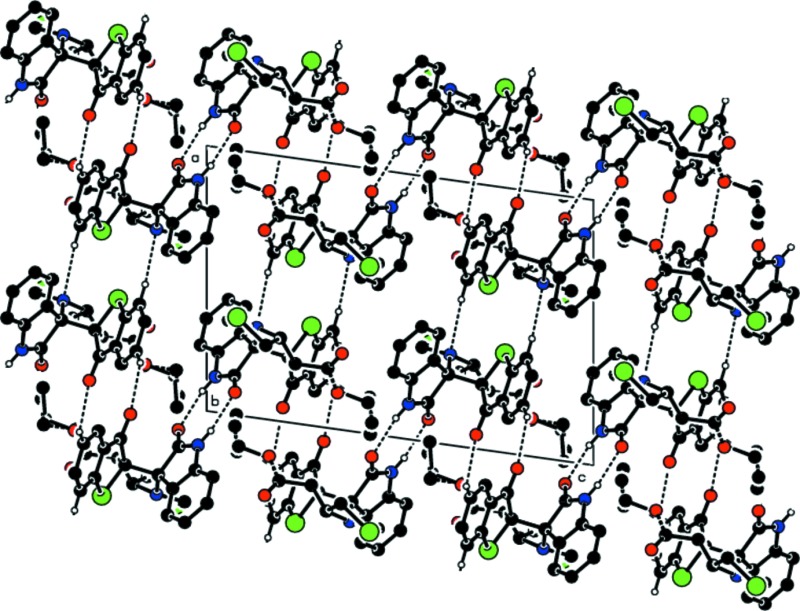
The molecular packing of the title compound viewed along the b axis. Dashed lines show the C—H···N, C—H···O and C—H···N hydrogen bonds (Table 1).

. DOI: 10.1107/S2056989015002030/su5067fig1.tif
The mol­ecular structure of the title compound with the atom numbering scheme. Displacement ellipsoids are drawn at the 30% probability level. The dashed lines represent the bonds involving the minor component of the disordered ethyl carboxyl­ate unit.

b . DOI: 10.1107/S2056989015002030/su5067fig2.tif
The mol­ecular packing of the title compound viewed along the *b* axis. Dashed lines show the C—H⋯N, C—H⋯O and C—H⋯N hydrogen bonds (Table 1).

CCDC reference: 1046458

Additional supporting information:  crystallographic information; 3D view; checkCIF report

## Figures and Tables

**Table 1:** Hydrogen-bond geometry (, )

*D*H*A*	*D*H	H*A*	*D* *A*	*D*H*A*
N2H2O4^i^	0.87(2)	2.05(2)	2.9029(18)	169(2)
C11H11O3^ii^	0.93	2.47	3.215(2)	137
C13H13N1^iii^	0.93	2.60	3.505(2)	165

Symmetry codes: (i) 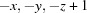; (ii) 
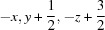
; (iii) 
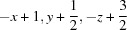
.

## supplementary crystallographic information

### S1. Comment

Spiro-pyrrolidine derivatives are unique tetracyclic 5-HT(2 A) receptor antagonists (Obniska *et al.*, 2003; Peddi *et al.*, 2004). These derivatives possess anticancer (Zapf *et al.*, 2011) and anti-influenza virus (Stylianakis *et al.*, 2003) activities. Highly functionalized pyrrolidines have gained much interest in the past few years as they constitute the main structural element of many natural and synthetic pharmacologically active compounds (Waldmann, 1995). Optically active pyrrolidines have been used as intermediates, chiral ligands or auxiliaries in controlled asymmetric synthesis (Suzuki *et al.*, 1994; Huryn *et al.*, 1991). In view of these importance and in continuation of our work on the crystal structure analysis of spiro-pyrrolidine derivatives, the crystal structure of the title compound has been carried out and the results are presented here.

The molecular structure of the title compound as illustrated in Fig. 1. The bond lengths and angles are within normal ranges and comparable to those found in a related structure (Narayanan *et al.*, 2013). Terminal atoms C1, C2, O2 in the ethyl carboxylate group are disordered over two positions [C1/C1A, C2/C2A & O2/O2A] with a site-occupancy ratio of 0.716 (12) : 0.284 (12). The sum of the angles at N1 and N2 [340.95° and 359.96°, respectively] of the pyrrolidine and indole rings are in accordance with sp^3^ and sp^2^ hybridization.

The central pyrrolidine ring (N1/C4-C7) forms dihedral angles of 82.0 (1)° and 80.9 (1)°, respectively, with pyrrolidine ring (N2/C6/C15-C16/C21) of indole ring system and the benzene ring (C9-C16) of benzothiophene ring system. The central pyrrolidine ring has an envelope conformation with atom C7 as the flap [puckering parameters of q_2_ = 0.4574 (2)Å , φ_2_ = 293.2 (2)°]. The thiophene ring (S1/C7-C9/C14) adopts twisted conformation on bond S1-C7 [puckering parameters of q_2_ = 0.1294 (2)Å, φ_2_ = 16.8 (8)°]. The five membered thiozole ring (N1/S2/C5/C22,C23) adopts twisted conformation on C23-S2 [puckering parameters of q_2_ = 0.4675 (2) Å, φ_2_ = 164.4 (2)°].

In the crystal, molecules are linked via C-H···O, C-H···N, N-H···O intermolecular hydrogen bonds (Table 1 and Fig. 2). The C11-H11···O3 inter-molecular interactions form a dimer with graphset motif R^2^_2_(12) and N2-H2···O4 hydrogen bonds connect molecules to form inversion dimers, with an R^2^_2_(8) ring motif (Fig. 2). C13-H13···N1 hydrogen bonds form a linear chain along the *a* axis. This combination of C-H···O, C-H···N and N-H···O hydrogen bonds gives a three-dimensional structure.

### S2. Experimental

A reaction mixture of (*E*)-ethyl 2-(3-oxobenzo[b]thiophen-2(3H)-ylidene) acetate (1.0 mmol), isatin (1.1 mmol) and thiazolidine-4-carboxylic acid (1.1 mmol) was refluxed in methanol (20 ml) until completion of the reaction monitored by TLC analysis. After completion of the reaction the solvent was evaporated under reduced pressure. The crude reaction mixture was dissolved in dichloromethane (2 × 50 ml) and washed with water followed by brine solution. The organic layer was separated and dried over sodium sulfate. After filtration the organic solvent was evaporated under reduced pressure. The product was separated by column chromatography using hexane and ethyl acetate (9:1) as an eluent to give a colourless solid. The product was dissolved in chloroform (3 ml) and heated for two minutes. The solution was then subjected to crystallization by slow evaporation of the solvent resulting in single crystals suitable for X-ray crystallographic studies.

### S3. Refinement

Atoms C1, C2 and O2 are disordered over two positions (C1A/C1B, C2A/C2B & O2/O2A) with a refined occupancy ratio of 0.716 (12) : 0.284 (12). The O-C and C-C distances of the disordered atoms were restrained to be equal. The displacement parameters of the disordered atoms were restrained to be equal for bonded atoms. The NH H atom was located in a difference Fourier map and freely refined. The C-bound H atoms were fixed geometrically and allowed to ride on their parent C atoms: C-H = 0.93-0.97 Å with *U*_iso_(H) = 1.5*U*_eq_(C) for methyl H atoms and = 1.2*U*_eq_(C) for other H atoms.

### Crystal data

**Table:**

C_23_H_20_N_2_O_4_S_2_	*F*(000) = 944
*M**_r_* = 452.53	*D*_x_ = 1.427 Mg m^−^^3^
Monoclinic, *P*2_1_/*c*	Mo *K*α radiation, λ = 0.71073 Å
Hall symbol: -P 2ybc	Cell parameters from 3706 reflections
*a* = 11.8894 (5) Å	θ = 2.3–25.0°
*b* = 10.2181 (4) Å	µ = 0.29 mm^−^^1^
*c* = 17.5044 (8) Å	*T* = 293 K
β = 97.991 (2)°	Block, colourless
*V* = 2105.91 (15) Å^3^	0.35 × 0.30 × 0.30 mm
*Z* = 4	

### Data collection

**Table:**

Bruker Kappa APEXII CCD diffractometer	3706 independent reflections
Radiation source: fine-focus sealed tube	3169 reflections with *I* > 2σ(*I*)
Graphite monochromator	*R*_int_ = 0.027
ω and φ scans	θ_max_ = 25.0°, θ_min_ = 2.3°
Absorption correction: multi-scan (*SADABS*; Bruker, 2004)	*h* = −14→14
*T*_min_ = 0.906, *T*_max_ = 0.919	*k* = −12→11
19635 measured reflections	*l* = −20→20

### Refinement

**Table:**

Refinement on *F*^2^	Primary atom site location: structure-invariant direct methods
Least-squares matrix: full	Secondary atom site location: difference Fourier map
*R*[*F*^2^ > 2σ(*F*^2^)] = 0.032	Hydrogen site location: inferred from neighbouring sites
*wR*(*F*^2^) = 0.088	H atoms treated by a mixture of independent and constrained refinement
*S* = 0.98	*w* = 1/[σ^2^(*F*_o_^2^) + (0.0443*P*)^2^ + 1.0594*P*] where *P* = (*F*_o_^2^ + 2*F*_c_^2^)/3
3705 reflections	(Δ/σ)_max_ < 0.001
312 parameters	Δρ_max_ = 0.26 e Å^−^^3^
65 restraints	Δρ_min_ = −0.27 e Å^−^^3^

### Special details

**Table:**

Geometry. All esds (except the esd in the dihedral angle between two l.s. planes) are estimated using the full covariance matrix. The cell esds are taken into account individually in the estimation of esds in distances, angles and torsion angles; correlations between esds in cell parameters are only used when they are defined by crystal symmetry. An approximate (isotropic) treatment of cell esds is used for estimating esds involving l.s. planes.
Refinement. Refinement of F^2^ against ALL reflections. The weighted R-factor wR and goodness of fit S are based on F^2^, conventional R-factors R are based on F, with F set to zero for negative F^2^. The threshold expression of F^2^ > 2sigma(F^2^) is used only for calculating R-factors(gt) etc. and is not relevant to the choice of reflections for refinement. R-factors based on F^2^ are statistically about twice as large as those based on F, and R- factors based on ALL data will be even larger.

### Fractional atomic coordinates and isotropic or equivalent isotropic displacement parameters (Å^2^)

**Table:**

	*x*	*y*	*z*	*U*_iso_*/*U*_eq_	Occ. (<1)
C3	0.22511 (16)	−0.15734 (19)	0.81332 (11)	0.0408 (4)	
C4	0.22481 (14)	−0.12481 (18)	0.72929 (10)	0.0342 (4)	
H4	0.1500	−0.1486	0.7019	0.041*	
C5	0.31386 (14)	−0.19721 (18)	0.69063 (10)	0.0354 (4)	
H5	0.3821	−0.2098	0.7283	0.042*	
C6	0.26623 (13)	0.00729 (17)	0.62479 (9)	0.0298 (4)	
C7	0.24619 (13)	0.02012 (17)	0.71114 (9)	0.0314 (4)	
C8	0.15041 (14)	0.11732 (18)	0.72277 (10)	0.0343 (4)	
C9	0.19513 (13)	0.23396 (18)	0.76473 (10)	0.0336 (4)	
C10	0.13286 (15)	0.3444 (2)	0.77968 (11)	0.0437 (5)	
H10	0.0549	0.3473	0.7640	0.052*	
C11	0.18760 (17)	0.4486 (2)	0.81770 (13)	0.0501 (5)	
H11	0.1467	0.5223	0.8285	0.060*	
C12	0.30442 (17)	0.4440 (2)	0.84011 (12)	0.0475 (5)	
H12	0.3411	0.5158	0.8650	0.057*	
C13	0.36685 (15)	0.33556 (19)	0.82629 (11)	0.0393 (4)	
H13	0.4450	0.3338	0.8414	0.047*	
C14	0.31142 (13)	0.22884 (17)	0.78951 (9)	0.0319 (4)	
C15	0.14879 (13)	−0.01360 (18)	0.57169 (9)	0.0334 (4)	
C16	0.22851 (14)	0.17108 (17)	0.52915 (10)	0.0331 (4)	
C17	0.24522 (16)	0.27754 (19)	0.48425 (11)	0.0415 (4)	
H17	0.1892	0.3057	0.4453	0.050*	
C18	0.34843 (18)	0.34133 (19)	0.49905 (12)	0.0461 (5)	
H18	0.3620	0.4140	0.4697	0.055*	
C19	0.43177 (17)	0.29921 (19)	0.55657 (12)	0.0451 (5)	
H19	0.5004	0.3441	0.5658	0.054*	
C20	0.41404 (15)	0.19029 (18)	0.60081 (11)	0.0381 (4)	
H20	0.4710	0.1606	0.6386	0.046*	
C21	0.31061 (13)	0.12661 (17)	0.58786 (10)	0.0309 (4)	
C22	0.35408 (17)	−0.17203 (19)	0.55696 (11)	0.0422 (4)	
H22A	0.4206	−0.1392	0.5368	0.051*	
H22B	0.2879	−0.1571	0.5189	0.051*	
N1	0.34078 (11)	−0.10643 (14)	0.62958 (8)	0.0313 (3)	
N2	0.13369 (12)	0.08759 (15)	0.52250 (9)	0.0373 (4)	
O1	0.28776 (14)	−0.23233 (15)	0.84987 (8)	0.0601 (4)	
O3	0.05293 (10)	0.09693 (14)	0.69704 (9)	0.0510 (4)	
O4	0.08550 (10)	−0.10764 (13)	0.57322 (7)	0.0430 (3)	
S1	0.37513 (3)	0.07922 (4)	0.77110 (3)	0.03457 (13)	
S2	0.36970 (5)	−0.34399 (5)	0.57908 (3)	0.05123 (16)	
C23	0.27897 (18)	−0.3280 (2)	0.65305 (13)	0.0504 (5)	
H23A	0.1995	−0.3274	0.6308	0.061*	
H23B	0.2917	−0.3989	0.6901	0.061*	
C1	−0.0015 (4)	−0.0975 (10)	0.9230 (3)	0.100 (2)	0.717 (12)
H1A	−0.0235	−0.1289	0.9704	0.149*	0.717 (12)
H1B	−0.0522	−0.1320	0.8803	0.149*	0.717 (12)
H1C	−0.0050	−0.0036	0.9220	0.149*	0.717 (12)
C2	0.1165 (5)	−0.1407 (8)	0.9171 (3)	0.0663 (17)	0.717 (12)
H2A	0.1212	−0.2354	0.9163	0.080*	0.717 (12)
H2B	0.1689	−0.1080	0.9603	0.080*	0.717 (12)
O2	0.1435 (9)	−0.0828 (9)	0.8414 (5)	0.059 (2)	0.717 (12)
C1A	0.0427 (18)	−0.1791 (17)	0.9270 (6)	0.085 (5)	0.283 (12)
H1A1	0.0230	−0.1767	0.9784	0.128*	0.283 (12)
H1A2	0.0661	−0.2661	0.9157	0.128*	0.283 (12)
H1A3	−0.0221	−0.1546	0.8908	0.128*	0.283 (12)
C2A	0.1380 (12)	−0.0854 (14)	0.9211 (8)	0.053 (3)	0.283 (12)
H2A1	0.1182	0.0052	0.9290	0.063*	0.283 (12)
H2A2	0.2080	−0.1085	0.9537	0.063*	0.283 (12)
O2A	0.1378 (19)	−0.120 (2)	0.8381 (10)	0.043 (3)	0.283 (12)
H2	0.0730 (15)	0.098 (2)	0.4891 (11)	0.051 (6)*	

### Atomic displacement parameters (Å^2^)

**Table:**

	*U*^11^	*U*^22^	*U*^33^	*U*^12^	*U*^13^	*U*^23^
C3	0.0357 (10)	0.0462 (11)	0.0390 (10)	−0.0027 (9)	−0.0008 (8)	0.0047 (9)
C4	0.0275 (8)	0.0386 (10)	0.0346 (9)	−0.0026 (7)	−0.0020 (7)	0.0035 (8)
C5	0.0308 (9)	0.0369 (10)	0.0369 (9)	−0.0002 (7)	−0.0010 (7)	0.0034 (8)
C6	0.0220 (8)	0.0339 (9)	0.0318 (9)	−0.0008 (7)	−0.0020 (6)	0.0000 (7)
C7	0.0209 (8)	0.0383 (10)	0.0330 (9)	−0.0002 (7)	−0.0032 (6)	−0.0002 (7)
C8	0.0235 (8)	0.0453 (10)	0.0335 (9)	0.0022 (7)	0.0016 (7)	0.0056 (8)
C9	0.0252 (8)	0.0411 (10)	0.0342 (9)	0.0037 (7)	0.0031 (7)	0.0033 (8)
C10	0.0270 (9)	0.0511 (12)	0.0526 (12)	0.0096 (8)	0.0035 (8)	0.0030 (9)
C11	0.0440 (11)	0.0426 (12)	0.0634 (13)	0.0125 (9)	0.0062 (10)	−0.0036 (10)
C12	0.0441 (11)	0.0392 (11)	0.0577 (12)	−0.0006 (9)	0.0015 (9)	−0.0070 (9)
C13	0.0291 (9)	0.0442 (11)	0.0432 (10)	0.0019 (8)	−0.0003 (8)	−0.0012 (9)
C14	0.0261 (8)	0.0375 (10)	0.0317 (8)	0.0034 (7)	0.0029 (7)	0.0027 (7)
C15	0.0257 (8)	0.0401 (10)	0.0325 (9)	0.0000 (7)	−0.0026 (7)	0.0007 (8)
C16	0.0326 (9)	0.0353 (10)	0.0314 (9)	0.0011 (7)	0.0041 (7)	−0.0025 (7)
C17	0.0473 (11)	0.0392 (11)	0.0377 (10)	0.0032 (8)	0.0049 (8)	0.0039 (8)
C18	0.0599 (12)	0.0340 (10)	0.0472 (11)	−0.0048 (9)	0.0168 (10)	0.0008 (9)
C19	0.0435 (11)	0.0407 (11)	0.0533 (12)	−0.0121 (9)	0.0148 (9)	−0.0078 (9)
C20	0.0298 (9)	0.0409 (10)	0.0433 (10)	−0.0028 (8)	0.0044 (7)	−0.0043 (8)
C21	0.0273 (8)	0.0317 (9)	0.0336 (9)	0.0007 (7)	0.0041 (7)	−0.0029 (7)
C22	0.0456 (11)	0.0405 (11)	0.0394 (10)	0.0036 (8)	0.0019 (8)	−0.0043 (8)
N1	0.0280 (7)	0.0332 (8)	0.0317 (7)	0.0019 (6)	0.0004 (6)	−0.0010 (6)
N2	0.0284 (8)	0.0437 (9)	0.0362 (8)	−0.0021 (6)	−0.0078 (6)	0.0061 (7)
O1	0.0696 (10)	0.0639 (10)	0.0447 (8)	0.0189 (8)	0.0010 (7)	0.0148 (7)
O3	0.0220 (6)	0.0628 (9)	0.0652 (9)	0.0014 (6)	−0.0046 (6)	−0.0071 (7)
O4	0.0315 (6)	0.0463 (8)	0.0463 (8)	−0.0113 (6)	−0.0115 (5)	0.0067 (6)
S1	0.0218 (2)	0.0417 (3)	0.0377 (2)	0.00479 (17)	−0.00465 (17)	−0.00587 (19)
S2	0.0606 (3)	0.0380 (3)	0.0542 (3)	0.0045 (2)	0.0047 (2)	−0.0082 (2)
C23	0.0546 (12)	0.0383 (11)	0.0580 (13)	−0.0051 (9)	0.0065 (10)	0.0005 (10)
C1	0.090 (3)	0.137 (6)	0.082 (3)	−0.018 (4)	0.047 (3)	−0.029 (4)
C2	0.068 (3)	0.093 (5)	0.041 (2)	−0.007 (3)	0.020 (2)	0.013 (3)
O2	0.060 (3)	0.076 (5)	0.0464 (19)	0.021 (3)	0.0200 (16)	0.014 (2)
C1A	0.123 (13)	0.102 (10)	0.039 (5)	−0.060 (9)	0.036 (6)	−0.012 (6)
C2A	0.062 (6)	0.050 (6)	0.046 (5)	−0.012 (5)	0.012 (4)	0.008 (5)
O2A	0.036 (4)	0.060 (8)	0.033 (4)	0.015 (5)	0.006 (3)	0.017 (4)

### Geometric parameters (Å, º)

**Table:**

C3—O1	1.191 (2)	C16—C21	1.392 (2)
C3—O2A	1.24 (2)	C16—N2	1.406 (2)
C3—O2	1.376 (10)	C17—C18	1.382 (3)
C3—C4	1.508 (2)	C17—H17	0.9300
C4—C5	1.525 (2)	C18—C19	1.380 (3)
C4—C7	1.543 (2)	C18—H18	0.9300
C4—H4	0.9800	C19—C20	1.389 (3)
C5—N1	1.483 (2)	C19—H19	0.9300
C5—C23	1.522 (3)	C20—C21	1.382 (2)
C5—H5	0.9800	C20—H20	0.9300
C6—N1	1.457 (2)	C22—N1	1.465 (2)
C6—C21	1.509 (2)	C22—S2	1.803 (2)
C6—C7	1.568 (2)	C22—H22A	0.9700
C6—C15	1.581 (2)	C22—H22B	0.9700
C7—C8	1.546 (2)	N2—H2	0.869 (15)
C7—S1	1.8355 (16)	S2—C23	1.804 (2)
C8—O3	1.202 (2)	C23—H23A	0.9700
C8—C9	1.461 (3)	C23—H23B	0.9700
C9—C14	1.391 (2)	C1—C2	1.488 (6)
C9—C10	1.394 (3)	C1—H1A	0.9600
C10—C11	1.372 (3)	C1—H1B	0.9600
C10—H10	0.9300	C1—H1C	0.9600
C11—C12	1.391 (3)	C2—O2	1.526 (9)
C11—H11	0.9300	C2—H2A	0.9700
C12—C13	1.373 (3)	C2—H2B	0.9700
C12—H12	0.9300	C1A—C2A	1.499 (9)
C13—C14	1.386 (3)	C1A—H1A1	0.9600
C13—H13	0.9300	C1A—H1A2	0.9600
C14—S1	1.7558 (17)	C1A—H1A3	0.9600
C15—O4	1.223 (2)	C2A—O2A	1.497 (17)
C15—N2	1.342 (2)	C2A—H2A1	0.9700
C16—C17	1.373 (3)	C2A—H2A2	0.9700
			
O1—C3—O2A	120.0 (9)	C18—C17—H17	121.2
O1—C3—O2	125.7 (4)	C19—C18—C17	121.24 (18)
O2A—C3—O2	16.2 (13)	C19—C18—H18	119.4
O1—C3—C4	125.62 (18)	C17—C18—H18	119.4
O2A—C3—C4	112.7 (8)	C18—C19—C20	120.51 (17)
O2—C3—C4	108.7 (4)	C18—C19—H19	119.7
C3—C4—C5	114.74 (15)	C20—C19—H19	119.7
C3—C4—C7	115.75 (15)	C21—C20—C19	119.12 (17)
C5—C4—C7	103.29 (13)	C21—C20—H20	120.4
C3—C4—H4	107.5	C19—C20—H20	120.4
C5—C4—H4	107.5	C20—C21—C16	118.96 (17)
C7—C4—H4	107.5	C20—C21—C6	131.79 (16)
N1—C5—C23	108.13 (15)	C16—C21—C6	109.21 (14)
N1—C5—C4	104.42 (14)	N1—C22—S2	106.25 (13)
C23—C5—C4	116.98 (15)	N1—C22—H22A	110.5
N1—C5—H5	109.0	S2—C22—H22A	110.5
C23—C5—H5	109.0	N1—C22—H22B	110.5
C4—C5—H5	109.0	S2—C22—H22B	110.5
N1—C6—C21	115.02 (13)	H22A—C22—H22B	108.7
N1—C6—C7	100.60 (12)	C6—N1—C22	116.95 (13)
C21—C6—C7	117.02 (14)	C6—N1—C5	110.48 (13)
N1—C6—C15	114.01 (13)	C22—N1—C5	113.52 (14)
C21—C6—C15	100.96 (13)	C15—N2—C16	112.36 (14)
C7—C6—C15	109.75 (12)	C15—N2—H2	123.3 (14)
C4—C7—C8	116.36 (14)	C16—N2—H2	124.3 (14)
C4—C7—C6	99.77 (13)	C14—S1—C7	92.53 (8)
C8—C7—C6	113.21 (13)	C22—S2—C23	90.69 (9)
C4—C7—S1	110.27 (11)	C5—C23—S2	103.62 (13)
C8—C7—S1	106.58 (12)	C5—C23—H23A	111.0
C6—C7—S1	110.61 (11)	S2—C23—H23A	111.0
O3—C8—C9	126.48 (17)	C5—C23—H23B	111.0
O3—C8—C7	121.94 (17)	S2—C23—H23B	111.0
C9—C8—C7	111.54 (13)	H23A—C23—H23B	109.0
C14—C9—C10	120.18 (17)	C2—C1—H1A	109.5
C14—C9—C8	113.73 (15)	C2—C1—H1B	109.6
C10—C9—C8	126.09 (15)	H1A—C1—H1B	109.5
C11—C10—C9	119.42 (17)	C2—C1—H1C	109.3
C11—C10—H10	120.3	H1A—C1—H1C	109.5
C9—C10—H10	120.3	H1B—C1—H1C	109.5
C10—C11—C12	119.88 (18)	C1—C2—O2	105.0 (7)
C10—C11—H11	120.1	C1—C2—H2A	110.6
C12—C11—H11	120.1	O2—C2—H2A	110.7
C13—C12—C11	121.40 (19)	C1—C2—H2B	110.8
C13—C12—H12	119.3	O2—C2—H2B	110.8
C11—C12—H12	119.3	H2A—C2—H2B	108.8
C12—C13—C14	118.87 (16)	C3—O2—C2	109.9 (7)
C12—C13—H13	120.6	C2A—C1A—H1A1	109.5
C14—C13—H13	120.6	C2A—C1A—H1A2	109.8
C13—C14—C9	120.21 (16)	H1A1—C1A—H1A2	109.5
C13—C14—S1	125.52 (13)	C2A—C1A—H1A3	109.1
C9—C14—S1	114.26 (13)	H1A1—C1A—H1A3	109.5
O4—C15—N2	126.28 (15)	H1A2—C1A—H1A3	109.5
O4—C15—C6	126.10 (15)	O2A—C2A—C1A	90.8 (14)
N2—C15—C6	107.56 (14)	O2A—C2A—H2A1	113.6
C17—C16—C21	122.63 (16)	C1A—C2A—H2A1	113.9
C17—C16—N2	127.72 (16)	O2A—C2A—H2A2	113.4
C21—C16—N2	109.58 (15)	C1A—C2A—H2A2	113.1
C16—C17—C18	117.51 (17)	H2A1—C2A—H2A2	110.8
C16—C17—H17	121.2	C3—O2A—C2A	121.6 (16)
			
O1—C3—C4—C5	0.2 (3)	C7—C6—C15—N2	118.46 (16)
O2A—C3—C4—C5	−165.0 (11)	C21—C16—C17—C18	0.1 (3)
O2—C3—C4—C5	178.2 (5)	N2—C16—C17—C18	−176.61 (17)
O1—C3—C4—C7	−120.0 (2)	C16—C17—C18—C19	0.3 (3)
O2A—C3—C4—C7	74.8 (11)	C17—C18—C19—C20	0.5 (3)
O2—C3—C4—C7	58.0 (5)	C18—C19—C20—C21	−1.7 (3)
C3—C4—C5—N1	−150.45 (14)	C19—C20—C21—C16	2.0 (3)
C7—C4—C5—N1	−23.56 (16)	C19—C20—C21—C6	179.27 (17)
C3—C4—C5—C23	90.12 (19)	C17—C16—C21—C20	−1.2 (3)
C7—C4—C5—C23	−142.99 (16)	N2—C16—C21—C20	175.96 (15)
C3—C4—C7—C8	−69.85 (19)	C17—C16—C21—C6	−179.05 (16)
C5—C4—C7—C8	163.90 (14)	N2—C16—C21—C6	−1.86 (19)
C3—C4—C7—C6	168.03 (14)	N1—C6—C21—C20	−49.8 (2)
C5—C4—C7—C6	41.78 (14)	C7—C6—C21—C20	68.0 (2)
C3—C4—C7—S1	51.64 (17)	C15—C6—C21—C20	−173.01 (18)
C5—C4—C7—S1	−74.60 (14)	N1—C6—C21—C16	127.64 (15)
N1—C6—C7—C4	−44.39 (14)	C7—C6—C21—C16	−114.60 (15)
C21—C6—C7—C4	−169.73 (13)	C15—C6—C21—C16	4.43 (17)
C15—C6—C7—C4	76.07 (15)	C21—C6—N1—C22	−69.72 (19)
N1—C6—C7—C8	−168.73 (13)	C7—C6—N1—C22	163.60 (14)
C21—C6—C7—C8	65.93 (18)	C15—C6—N1—C22	46.2 (2)
C15—C6—C7—C8	−48.27 (18)	C21—C6—N1—C5	158.38 (13)
N1—C6—C7—S1	71.74 (13)	C7—C6—N1—C5	31.70 (15)
C21—C6—C7—S1	−53.60 (16)	C15—C6—N1—C5	−85.67 (16)
C15—C6—C7—S1	−167.80 (11)	S2—C22—N1—C6	−145.63 (12)
C4—C7—C8—O3	−48.7 (2)	S2—C22—N1—C5	−15.15 (17)
C6—C7—C8—O3	66.1 (2)	C23—C5—N1—C6	119.60 (16)
S1—C7—C8—O3	−172.12 (15)	C4—C5—N1—C6	−5.64 (17)
C4—C7—C8—C9	133.65 (15)	C23—C5—N1—C22	−14.04 (19)
C6—C7—C8—C9	−111.60 (16)	C4—C5—N1—C22	−139.28 (14)
S1—C7—C8—C9	10.22 (17)	O4—C15—N2—C16	−172.24 (17)
O3—C8—C9—C14	178.35 (18)	C6—C15—N2—C16	5.08 (19)
C7—C8—C9—C14	−4.1 (2)	C17—C16—N2—C15	174.80 (18)
O3—C8—C9—C10	−2.6 (3)	C21—C16—N2—C15	−2.2 (2)
C7—C8—C9—C10	174.91 (17)	C13—C14—S1—C7	−171.66 (16)
C14—C9—C10—C11	1.2 (3)	C9—C14—S1—C7	9.31 (14)
C8—C9—C10—C11	−177.76 (18)	C4—C7—S1—C14	−137.86 (12)
C9—C10—C11—C12	0.7 (3)	C8—C7—S1—C14	−10.72 (12)
C10—C11—C12—C13	−1.2 (3)	C6—C7—S1—C14	112.74 (12)
C11—C12—C13—C14	−0.3 (3)	N1—C22—S2—C23	31.91 (14)
C12—C13—C14—C9	2.3 (3)	N1—C5—C23—S2	36.32 (17)
C12—C13—C14—S1	−176.71 (15)	C4—C5—C23—S2	153.75 (13)
C10—C9—C14—C13	−2.7 (3)	C22—S2—C23—C5	−39.29 (14)
C8—C9—C14—C13	176.35 (16)	O1—C3—O2—C2	−19.0 (10)
C10—C9—C14—S1	176.34 (14)	O2A—C3—O2—C2	56 (4)
C8—C9—C14—S1	−4.56 (19)	C4—C3—O2—C2	163.0 (6)
N1—C6—C15—O4	47.7 (2)	C1—C2—O2—C3	−154.3 (7)
C21—C6—C15—O4	171.64 (17)	O1—C3—O2A—C2A	42 (2)
C7—C6—C15—O4	−64.2 (2)	O2—C3—O2A—C2A	−73 (4)
N1—C6—C15—N2	−129.59 (15)	C4—C3—O2A—C2A	−151.8 (15)
C21—C6—C15—N2	−5.69 (17)	C1A—C2A—O2A—C3	−126.0 (17)

### Hydrogen-bond geometry (Å, º)

**Table:**

*D*—H···*A*	*D*—H	H···*A*	*D*···*A*	*D*—H···*A*
N2—H2···O4^i^	0.87 (2)	2.05 (2)	2.9029 (18)	169 (2)
C11—H11···O3^ii^	0.93	2.47	3.215 (2)	137
C13—H13···N1^iii^	0.93	2.60	3.505 (2)	165

Symmetry codes: (i) −*x*, −*y*, −*z*+1; (ii) −*x*, *y*+1/2, −*z*+3/2; (iii) −*x*+1, *y*+1/2, −*z*+3/2.
