# Supplementary figures and images for: Crystal structure of ethyl 2′′,3-dioxo-7′,7a’-di­hydro-1′H,3H,3′H-di­spiro[benzo[b]thio­phene-2,6′-pyrrolo­[1,2-c]thia­zole-5′,3′′-indoline]-7′-carboxyl­ate
Source: Acta Crystallogr E Crystallogr Commun. 2015 Feb 7;71(Pt 3):o148–9. doi: 10.1107/S2056989015002030 (PMC4350756; doi:10.1107/S2056989015002030)

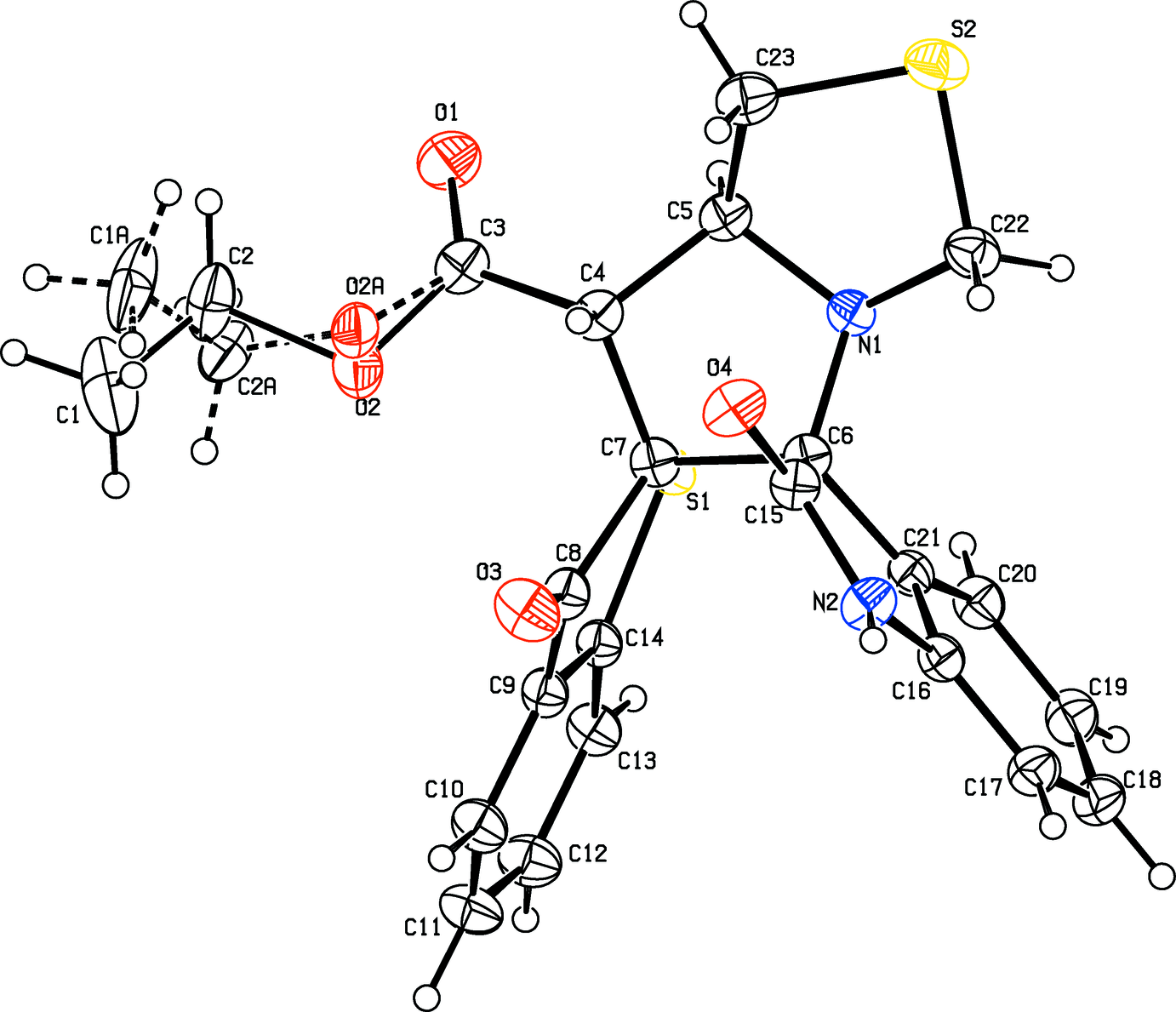

Supplement: Supplementary file 3 [file e-71-0o148-fig1.tif]

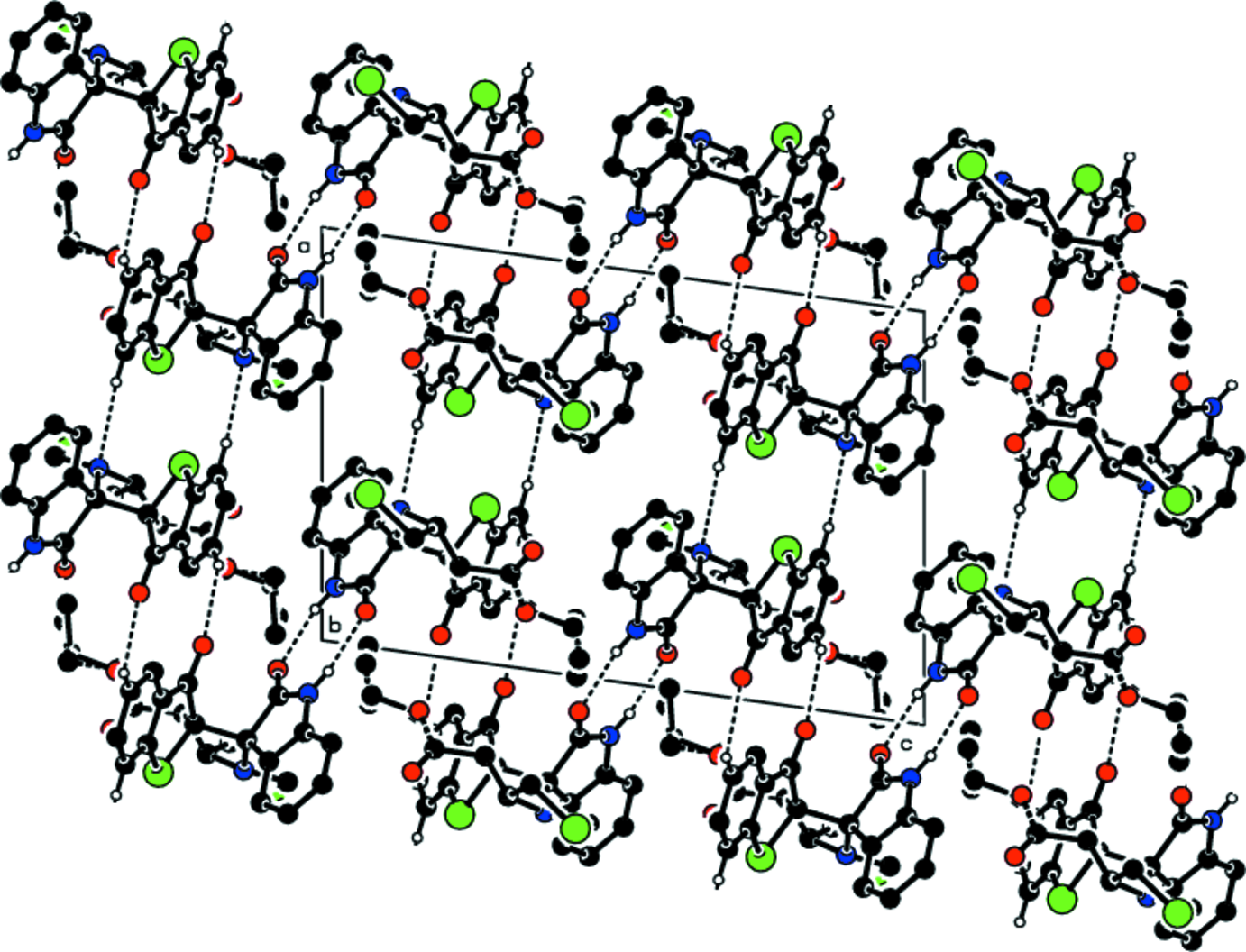

Supplement: Supplementary file 4 [file e-71-0o148-fig2.tif]
